# Risk effects of near-roadway pollutants and asthma status on bronchitic symptoms in children

**DOI:** 10.1097/EE9.0000000000000012

**Published:** 2018-04-30

**Authors:** Robert Urman, Sandrah Eckel, Huiyu Deng, Kiros Berhane, Ed Avol, Fred Lurmann, Rob McConnell, Frank Gilliland

**Affiliations:** a Department of Preventive Medicine, Keck School of Medicine, University of Southern California, Los Angeles, California; b Sonoma Technology, Inc., Petaluma, California.

## Abstract

**Background:**

Bronchitic symptoms in children pose a significant clinical and public health burden. Exposures to criteria air pollutants affect bronchitic symptoms, especially in children with asthma. Less is known about near-roadway exposures.

**Methods:**

Bronchitic symptoms (bronchitis, chronic cough, or phlegm) in the past 12 months were assessed annually with 8 to 9 years of follow-up on 6757 children from the southern California Children’s Health Study. Residential exposure to freeway and non-freeway near-roadway air pollution was estimated using a line-source dispersion model. Mixed-effects logistic regression models were used to relate near-roadway air pollutant exposures to bronchitic symptoms among children with and without asthma.

**Results:**

Among children with asthma, a 2 SD increase in non-freeway exposures (odds ratio [OR]: 1.44; 95% confidence interval [CI]: 1.17, 1.78) and freeway exposures (OR: 1.31; 95% CI: 1.06, 1.60) were significantly associated with increased risk of bronchitic symptoms. Among children without asthma, only non-freeway exposures had a significant association (OR: 1.14; 95% CI: 1.00, 1.29). Associations were strongest among children living in communities with lower regional particulate matter.

**Conclusions:**

Near-roadway air pollution was associated with bronchitic symptoms, especially among children with asthma and those living in communities with lower regional particulate matter. Better characterization of traffic pollutants from non-freeway roads is needed since many children live in close proximity to this source.

## Abstract 2

Supplemental Digital Content is available in the text.

**What This Study Adds**

Near-roadway air was associated with an increased risk of bronchitic symptoms in adolescents, especially among those having asthma. These associations with were near-roadway pollution more pronounced in communities that had lower concentrations of regional fine particulate matter, suggesting that even in communities that appear to be “cleaner,” there are some individuals who may be at greater risk than others.

## Introduction

Bronchitic symptoms in children, especially among those diagnosed with asthma, represent a significant burden to both clinical and public health that also results in reduced quality of life and considerable economic costs.^1,2^ Several studies have now shown positive associations between regional air pollutants and prevalence of bronchitic symptoms in children.^3–8^ Moreover, recent findings from the southern California Children’s Health Study (CHS) suggested that reductions in ambient pollution levels across several cohorts of children were associated with lower prevalence of bronchitic symptoms, further implicating the importance of air pollution in the etiology of this outcome.^9^

Identifying specific components of the air pollution mixture responsible for observed health effects has been of both academic and regulatory interest. In a previous CHS study, several components of regional pollution were found to be associated with bronchitic symptoms including nitrogen dioxide (NO_2_) and fine particulate matter (PM_2.5_) as well as constituents of PM_2.5_ such as elemental carbon (EC) and organic carbon.^4^ In southern California, traffic represents the primary source of many of these air pollutants.^10^ Although reductions in regional air pollution in southern California have taken place over past decades, the number of vehicles and vehicle miles driven have increased.^11^ Much less is known about whether exposure to air pollutants from near-roadway sources is associated with bronchitic symptoms in children. These near-roadway pollutants include oxides of nitrogen (NO_x_), ultrafine particles, carbon monoxide (CO), EC, and gaseous and particle phase organic compounds such as polycyclic aromatic hydrocarbons, all of which are corre lated and decay sharply with increasing distance away from a roadway.^12–15^ Because exposure to vehicle emissions from freeways and non-freeway roads are likely to be different mixtures because of differences in proportion of heavy-duty trucks, vehicle speed, traffic volumes, and frequency of acceleration, braking, and cold starts,^15–17^ we investigated whether the associations varied for exposures from freeways and non-freeway roads. Furthermore, since previous studies have shown that regional air pollution is strongly associated with bronchitic symptoms, and traffic is an important contributor to regional pollution in southern California, we were interested in whether the effect of near-roadway air pollution (NRAP) differed across communities with higher and lower levels of regional pollution.

Understanding variation in susceptibility to the effect of air pollutant exposure is important for clinical and regulatory decision making. Substantial evidence supports increased susceptibility in children with asthma.^18^ In the current longitudinal study, we investigated whether exposure to NRAP from both freeways and non-freeway roads were associated with increased risk of bronchitic symptoms across the study follow-up period in three cohorts of school-aged children in the CHS. We hypothesized that the effect of traffic would be larger among children diagnosed with asthma.

## Methods

### Study participants

**Figure 1.**
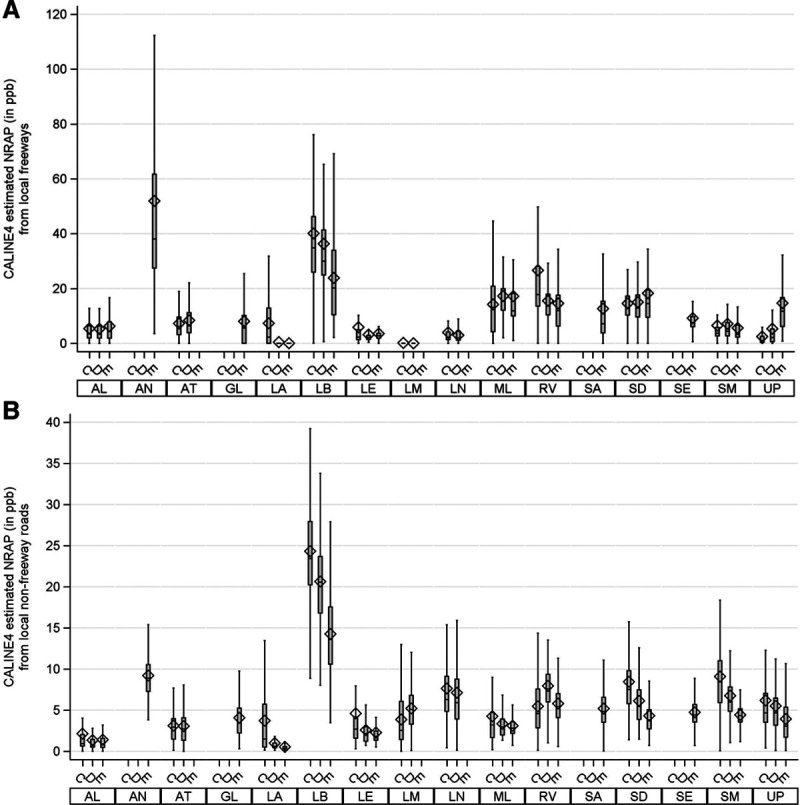
Distribution of residential estimates of (A) freeway and (B) non-freeway exposures of near-roadway air pollution (NRAP) from the California Line Source Dispersion Model (CALINE4) by community* and cohort†. Estimates of NRAP are scaled to vehicle emission rates of oxides of nitrogen (NO_x_) and reported in parts per billion. *Community abbreviations: AL indicates Alpine; AN, Anaheim; AT, Atascadero; GL, Glendora; LA, Lake Arrowhead; LB, Long Beach; LE, Lake Elsinore; LM, Lompoc; LN, Lancaster; ML, Mira Loma; RV, Riverside; SA, Santa Barbara; SD, San Dimas; SE, San Bernardino; SM, Santa Maria; UP, Upland. †Participants from Cohorts C, D, and E were followed from 1993 to 2001, 1996 to 2004, and 2002 to 2010, respectively.

Children across 16 primarily urban Southern California communities from the three most recent cohorts participating in the CHS were selected for this analysis. Students were recruited from fourth grade classrooms (≈9 years of age) in 1993 and 1997 (hereafter referred to as Cohorts C [N = 1800] and D [N = 2080], respectively) and followed through 18 years of age across 12 communities, while the third cohort was composed of students recruited from kindergarten and first grade classrooms (≈5–6 years of age) in 2003 (hereafter referred to as Cohort E [N = 5600]) and followed through 16 years of age across 13 communities; nine of the communities overlapped across all three cohorts (see Figure 1). Annual questionnaires were administered to parents and students to assess the children’s respiratory health, asthma medication use, and other important related information including the social and physical environment of the home.^9^

### Bronchitic symptoms and asthma status

Chronic bronchitic symptoms in the prior 12 months were assessed at each annual visit over an 8- to 9-year period (median follow-up: 7 years), as previously described.^3,9^ A child was considered to have had chronic bronchitic symptoms if there was at least one positive report in the past 12 months of (1) a daily cough for 3 months in a row, (2) congestion or phlegm other than when accompanied by a cold, or (3) bronchitis. Children from Cohorts C and D were considered to have a history of asthma by 10 years of age if there was a “yes” response to the question, “Has a doctor ever diagnosed this child as having asthma?” on the baseline questionnaire. Because children in Cohort E entered the study at a younger age (5–7 years of age), a child was considered to have a history of asthma by 10 years of age if there was a “yes” response to the same question at any time during follow-up up through age 10 years. Children without asthma were those who never reported having asthma before or during any period of follow-up.

### Near-roadway air pollution

Residential exposure to the NRAP mixture from freeways and other non-freeway roads located within 5 km from their residences was estimated using the California Line Source Dispersion Model (CALINE4).^19^ The CALINE4 model used distance to roadways, vehicle counts, vehicle emission rates, and meteorological conditions as inputs. This model accounts for the higher vehicle speeds, fraction of heavy-duty trucks, and traffic volumes on freeways than on non-freeways. It does not account for the enhanced acceleration, braking, and cold starts that occurs on non-freeways compared to freeways. We chose to use NO_x_ as a surrogate for the near-roadway mixture because the emission factor model (EMFAC2014) produces more accurate vehicle emission rates for NO_x_ than other species (PM, CO, or non-methane hydrocarbons).^20,21^ While NO_x_ was selected as the surrogate to represent this mixture, we will refer to the output from these models as NRAP from freeways and non-freeways. Modeled NRAP, estimated as a time-varying annual average, were lagged a year to temporally align exposures with bronchitic symptoms from the prior 12 months. Total NRAP was computed as the sum of NRAP from freeways and non-freeways.

### Data analysis

The primary analyses were restricted to 6757 children with and without asthma who were (1) diagnosed by the age of 10 years and (2) had at least two assessments of bronchitic symptoms, for consistency with a recently published study.^9^ We excluded children whose baseline asthma status was unknown or who developed asthma after 10 years of age so that we could focus on the association of near-roadway pollutants with bronchitic symptoms in children with and without asthma rather than attempting to address the issue of whether exposure to near-roadway pollutants is associated with incident asthma and then bronchitic symptoms in these incident cases. Multilevel logistic models were used to examine the associations of NRAP from freeways and non-freeway roads with bronchitic symptoms in these longitudinal analyses. A random intercept for individuals was included to account for serial dependency across observations from the same individual. Models included fixed effects for sex, ethnicity, and age. Models were adjusted for community to focus the associations on within community comparisons and to control for any between community differences (but hence also precluding the joint analysis of near-roadway and regional pollutants). Models were also adjusted for cohort to focus the associations on within-cohort comparisons and to control for any long-term temporal changes in exposure, outcome, and potential confounders. Effect estimates were separately scaled to 2 SD of the total distribution of NRAP from either freeways or non-freeway roads across all individuals. Separate estimates for children with asthma and children without asthma were computed by including an interaction term between asthma status and exposure. Associations were also reported by higher and lower levels of regional PM_2.5_ and ozone. This was accomplished by taking the median of all available annual concentrations at each community’s regional monitor during the study period and then dichotomizing these communities into “higher” and “lower” PM_2.5_ or ozone communities (See eFigure 1; http://links.lww.com/EE/A7). An interaction term between these indicators and exposure to NRAP was then included in the model to obtain separate estimates of NRAP in each of these categories of regional pollutants. Regional NO_2_ was also considered, but the communities sorted into the same higher and lower categorizations as PM_2.5_. Several sensitivity analyses were conducted, including adjustment for additional covariates (parental education [marker of socioeconomic status]); use of asthma medication; presence in the home of roaches, dogs, cats, gas stove, mildew, or second-hand smoke; and participant’s overweight/obesity status), restricting data to participants with observations in the first and last years of follow-up, and including participants who developed asthma after the age of 10 years.

All analyses assumed a two-sided alternative hypothesis at a 0.05 level of significance. All models were fitted using SAS version 9.4 (SAS Institute Inc, Cary, NC).

## Results

Among the 6757 participants who were included in the primary analyses, about half were females (see Table 1). Hispanic White participants made up about 46% of the population and Non-Hispanic White participants made up another 41% of the population. Approximately 20% of the participants were diagnosed with asthma by 10 years of age. Among children with asthma, the average prevalence of bronchitic symptoms across all ages was about 35%, while among children without asthma, the prevalence was around 15%. The distributions of bronchitic symptoms across age by asthma status and cohort are shown in eFigure 2; http://links.lww.com/EE/A7. The associations of child ethnicity and parental education with bronchitic symptoms differed by childhood asthma status (see eTable 1; http://links.lww.com/EE/A7).

**Table 1:**
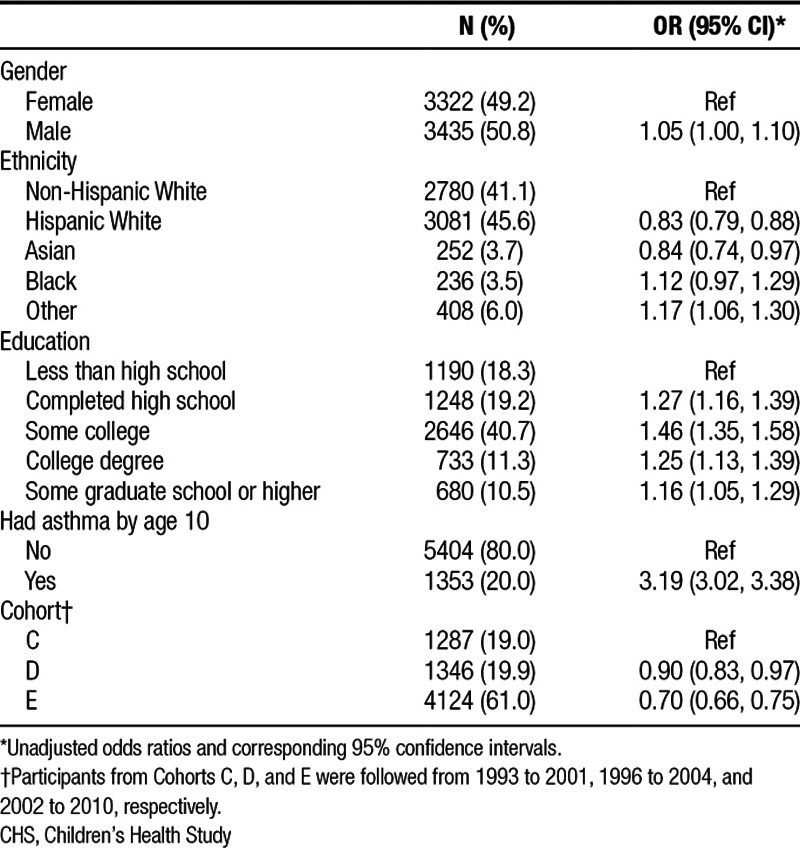
Distribution of demographic characteristics among CHS study participants and their association with bronchitic symptoms

The mean concentration of NRAP from freeways was highest in Cohort E, partly because of the inclusion of Anaheim as a study community, which had the highest concentrations across all communities (see Figure 1 and Table 2). There was substantial within-community variability in freeway exposures in Anaheim and Long Beach; participants in Lake Arrowhead (Cohorts D and E) and Lompoc (Cohorts C and D) lived too far away from freeways to have quantifiable freeway exposures. Levels of NRAP from non-freeway roads were highest in Cohort C for most communities, and Long Beach had the highest average NRAP levels compared to the other communities. On average, among all participants across all communities and cohorts, 56% of CALINE4 estimates for total NRAP were attributed to NRAP from freeways (range: 1% to 81%).

**Table 2:**
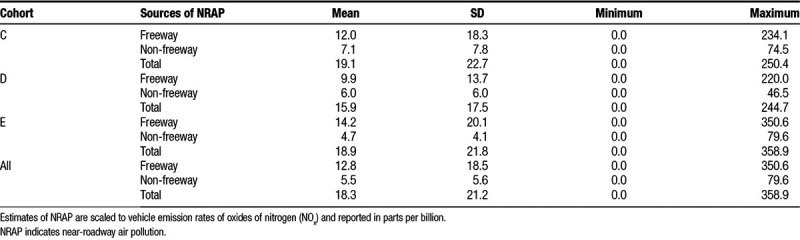
Descriptive summary of NRAP

Exposure to NRAP was associated with bronchitic symptoms (Table 3). Among all participants, a 2 SD increase in NRAP from non-freeway roads was associated with an increased odds of bronchitic symptoms (OR: 1.18; 95% CI: 1.04, 1.33). This association between NRAP from non-freeway roads and bronchitic symptoms was more pronounced among children with asthma (OR: 1.44; 95% CI: 1.17, 1.78) compared to children without asthma (OR: 1.14; 95% CI: 1.00, 1.29). Exposure to NRAP from freeways was not associated with bronchitic symptoms in the group of all participants combined; however, children with asthma showed greater susceptibility. Among children with asthma, a 2 SD increase in NRAP from freeways was associated with a 31% increase in the odds of bronchitic symptoms (OR: 1.31; 95% CI: 1.06, 1.60); no associations were observed among children without asthma. The associations of NRAP from freeways and non-freeways were qualitatively similar in models where they were entered jointly (see eTable 2; http://links.lww.com/EE/A7). Associations of bronchitic symptoms with total NRAP exposure were similar to that of NRAP from freeways.

**Table 3:**
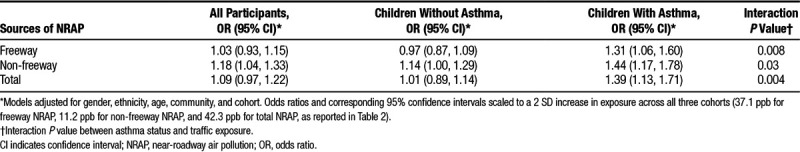
Effect of NRAP on bronchitic symptoms by asthma status

Differences in associations between NRAP and bronchitic symptoms were observed by community levels of regional PM_2.5_. Among all participants living in communities with lower regional PM_2.5_, a 2 SD increase in total NRAP exposure was associated with a 46% increase in the odds of bronchitic symptoms (OR: 1.46; 95% CI: 1.10, 1.94; see Table 4), but little evidence for associations was observed among those living in higher PM_2.5_ communities (OR: 1.02; 95% CI: 0.90, 1.16; *P* value for interaction: 0.02). Associations of bronchitic symptoms with NRAP from freeways were similar to those of total NRAP exposure in lower and higher PM_2.5_ communities, but the difference in associations was only marginally significant (*P* value: 0.05).

**Table 4:**
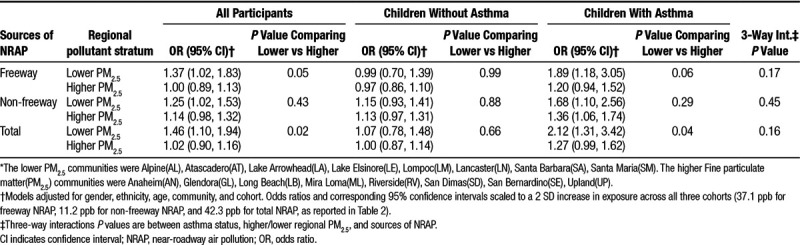
Effect of NRAP on bronchitic symptoms by asthma status and levels of regional PM_2.5_*

These differences in NRAP associations with bronchitic symptoms between lower and higher PM_2.5_ communities among all participants were driven mainly by children with asthma. The prevalence of asthma among participants in communities with lower PM_2.5_ was 18% compared to 21% of participants in communities with higher PM_2.5_. Children with asthma living in lower PM_2.5_ communities were at 89% increased odds of reporting bronchitic symptoms in the past 12 months per 2 SD increase in NRAP from freeways (OR: 1.89; 95% CI: 1.18, 3.05; see Table 4). Those living in higher PM_2.5_ communities did not have a significantly higher odds of bronchitic symptoms for the same increase in exposure to freeway pollutants (OR: 1.20; 95% CI: 0.94, 1.52; *P* value for difference: 0.06). Associations between non-freeways exposures and bronchitic symptoms were observed among children with asthma in both lower and higher PM_2.5_ communities, but the difference in magnitude was not statistically different. There was no significant difference in effects of NRAP by higher and lower ozone levels (see eTable 3; http://links.lww.com/EE/A7).

Results were generally robust to a number of sensitiv ity analyses (eTable 4; http://links.lww.com/EE/A7). Stronger associations were observed when analyses were restricted to participants who completed follow-up to the last study visit. Associations were also robust to adjustment for several potential confounders (e.g., second-hand smoke, medication use, and indoor exposures; see Table 4 for full list). In sensitivity analyses analyzing all subjects that also included subjects who developed asthma after 10 years of age and subjects with only single measurements of bronchitic symptoms, associations were attenuated but remained statistically significant.

## Discussion

In this longitudinal study of school-aged children, exposure to pollutants from local freeways and non-freeway roads was associated with increased risk of having bronchitic symptoms. The strength of these associations differed by asthma status and by community of residence background regional pollution. Children with asthma appeared to be more susceptible to freeway-related air pollutants because it was only among these participants where we saw a positive association between freeway-related air pollutants and bronchitic symptoms, whereas positive associations of bronchitic symptoms with pollutants from non-freeway roads were observed among all participants. These associations were robust in several sensitivity analyses and after adjustment for potential confounders.

The near-roadway pollutant mixture is composed of particles and gases. Ambient measures of pollutants include both direct emissions from traffic as well as secondary compounds formed as a result of atmospheric photochemistry and regional transport. In previous CHS studies, we have shown associations between different measures of ambient pollutants (NO_2_, PM_2.5_), with increased reports of bronchitic symptoms.^3,4^ Moreover, EC, a constituent of PM_2.5_ and a known marker of diesel emissions, was also associated with bronchitic symptoms.^4^ Several other studies have shown associations between higher levels of traffic-related air pollutants, including NO_2_, PM_2.5_ and EC, with increased frequency of respiratory outcomes such as bronchitis, cough, wheeze, and chest tightness, as well as hospital admission owing to respiratory morbidities.^22–28^

Exposure to near-roadway pollutants as assessed by statistical modeling, intense measurements of pollutants near roadways, or using surrogate measures such as proximity to roadways or traffic density on nearby roads have all been shown to be associated with several respiratory outcomes in children, including decreased lung function and increased risk of asthma, wheeze, and cough.^22,29–31^ Additionally, several cross-sectional studies using these measures have also been shown to be associated with increased risk of bronchitis in children,^29,32,33^ while a case–control study among infants found some markers of traffic to be associated with an increased risk of bronchiolitis.^34^ However, a Dutch study found no significant associations between modeled traffic pollutants and bronchitis among children but did find suggestive associations with other outcomes, including wheezing and asthma.^35^

A common proposed mechanism by which these near-roadway pollutants affect respiratory health in children is through airway inflammation. There is evidence suggesting that exposure to these pollutants can induce oxidative stress in the lungs, resulting in airway inflammation.^36,37^ Children with asthma may be particularly sensitive to the effects of air pollution because of lower levels of antioxidants in the lung lining fluid.^38^ For example, a recent study from the CHS found that the length of road near the residence was associated with higher airway inflammation as measured using exhaled nitric oxide in children with asthma; no associations were observed in children without asthma.^39^ One study showed that exposure to diesel exhaust particles was associated with an increase in airway responsiveness among adults with asthma.^40^ Thus, the strong association of NRAP with bronchitic symptoms among children with asthma may be capturing exacerbations of asthma as asthma and bronchitic symptoms are often correlated.^41,42^

While associations with bronchitic symptoms were observed with exposure to pollutants from freeways and non-freeways among children with asthma, only exposure to pollutants from non-freeway roads was associated with bronchitic symptoms among children with no asthma. The relatively weaker associations with exposure to pollutants from freeways was not expected, given the higher concentrations of predicted pollutants from freeways compared to non-freeway roads observed in our data. However, these findings are consistent with another study from the CHS that found pollutants from non-freeway roads to be more strongly associated with asthma incidence than pollutants from freeways.^43^

It is unclear as for the stronger associations of bronchitic symptoms with non-freeway pollutants. CALINE4 estimates of pollutants from non-freeway roads may have been estimated with more error compared to those from freeway roads because of the likelier smaller spatial scale of pollutants from the large network of non-freeway roads and less reliable input data regarding traffic flow on these particular roads. However, the misclassification of non-freeway exposures with respect to bronchitic symptoms is likely to be nondifferential, which would cause a bias of the observed effects toward the null and an underestimation of the true effect. One possible explanation for the difference in associations may be explained by the non-freeway emission mixture being likely different than that from freeways. The fraction of heavy-duty diesel trucks is higher on freeways than on non-freeways in Southern Californian, and a Californian study has shown that the chemical composition of compression-ignition (diesel) engine emissions and spark ignition (gasoline) engine emissions are quite different.^44^ Another study in Texas found notable differences in PM composition, ultrafine particle number, and gas concentrations upwind and downwind of (1) a major non-freeway with primarily passenger vehicles, (2) a limited access freeway, and (3) a heavily traveled non-freeway with a high proportion of truck traffic.^15,16^ For example, numerous polycyclic aromatic hydrocarbons, formaldehyde, acetaldehyde, and acrolein had higher concentrations 35 m downwind of the major non-freeway than 40 m downwind of the Interstate freeway that had more than double the traffic volume.^13^ Cold starts, which are more likely to occur near residences and thus non-freeway roads, result in higher levels of pollutants generated because of catalytic converters having not reached optimal temperature to efficiently reduce emissions.^45^ Another study found that PM concentrations were higher during delay periods at signalized traffic intersections compared to free-flowing traffic.^17^ More research is needed to better characterize differences in near-roadway pollutants from freeways and non-freeway roadways and to determine whether differences in composition may explain the difference in observed associations.

In two previously reported studies from the CHS, both regional and NRAP were important determinants of respiratory outcome.^30,46^ In the current study, reported associations between near-roadway pollutants and bronchitic symptoms were weakest in communities that historically had higher levels of PM_2.5_ (and NO_2_) and strongest in those with lower concentrations. One possible explanation is that the effect of NRAP is masked in communities with higher regional PM_2.5_ and NO_2_. In other words, a possible dose–response effect may be occurring where higher regional pollution may have already resulted in an exacerbation of symptoms among children with asthma, so the effect of NRAP is smaller on a relative scale, given a high background risk. Thus, in communities with lower PM_2.5_ and NO_2_, the effect of NRAP would be more apparent.

A limitation of this study was the use of estimated measures of residential near-roadway exposure based on the CALINE4 model, which may not capture true personal exposures and thus lead to some exposure misclassification. However, this exposure metric has been previously shown to capture adequately the local scale variability of traffic sources in a majority of these study communities in two separate exposure assessment campaigns.^10,47^ Another limitation was the use of self-report for case ascertainment of bronchitic symptoms in the past 12 months and for asthma status. Self-report of asthma status has been validated in a subset of participants based on review of medical records, thus, lending credibility to our assessment of asthma status.^48^ Bronchitic symptoms in this study was defined based on at least one affirmation of either a cough that last for at least 3 months, chronic phlegm, or a report of diagnosis. These are all symptoms that are likely to be remembered well.^4^ However, misclassification of both bronchitic symptoms and asthma may still have occurred. Any misclassification of bronchitic symptoms or asthma is unlikely to be related to the model-based assignment of exposures, which would result in more conservative effect estimates.

This study also has a number of strengths. The longitudinal assessment of bronchitic symptoms among three cohorts of youth across several southern Californian communities with a wide range of traffic and regional air pollutant exposures, spanning a 20-year period, is a major and obvious strength. The use of a population-based sample of participants from a number of schools allows for increased generalizability of findings to other youth in this region. Another strength was the consistent collection of important covariates allowing for adjustment for several factors that could confound the observed relationship.

This is one of the first studies to report associations of near-roadway pollutants with repeated measures of bronchitic symptoms on the same participant. These findings provide further evidence of the potential harm of NRAP, especially among children with asthma. More studies are needed to determine what components of NRAP are likely to induce the observed associations. Additional work is also needed to better understand the variations in effects of NRAP in lower and higher PM_2.5_ communities.

## Conflict of interest statement

Fred Lurmann is employed by Sonoma Technology, Inc (Petaluma, CA). All other authors declare they have no actual or potential competing interests beyond grant support from the NIH and the South Coast Air Quality Management District. This work was supported by the National Institutes of Health (grant nos. P30ES007048, P01ES009581, P01ES011627, P01ES022845, R01 ES016535, R03ES014046, P50 CA180905, R01HL061768, R01HL076647, R01HL087680, and RC2HL101651), the Environmental Protection Agency (grant nos. RD83544101, R826708, RD831861, and R831845), the South Coast Air Quality Management District (Personalized Strategies to Prevent Air Pollution-Induced Asthma Exacerbations [RFP no. PBOC-9]), and the Hastings Foundation. Because of the limitations in the original consent forms and HIPAA requirements, the data from the Southern California Children Health Study (CHS) cannot be freely available in the manuscript, supplemental files, or in a public repository. However, we are committed to sharing the data and results acquired as part of this study. The CHS has a process in place for data sharing that involves approval of proposals by a Data Sharing Committee composed of USC and California ARB selected academic members. Investigators who want access to data will be required to submit a brief research protocol, which will be reviewed by the Children’s Health Study Health Data Release Committee and the USC IRB. Please send requests to access this dataset to the corresponding author, Dr. Frank Gilliland (gillilan@usc.edu).
